# Supplementary figures and images for: Comparative proteomic analysis of pepper (Capsicum annuum L.) seedlings under selenium stress
Source: PeerJ. 2019 Nov 27;7:e8020. doi: 10.7717/peerj.8020 (PMC6884995; doi:10.7717/peerj.8020)

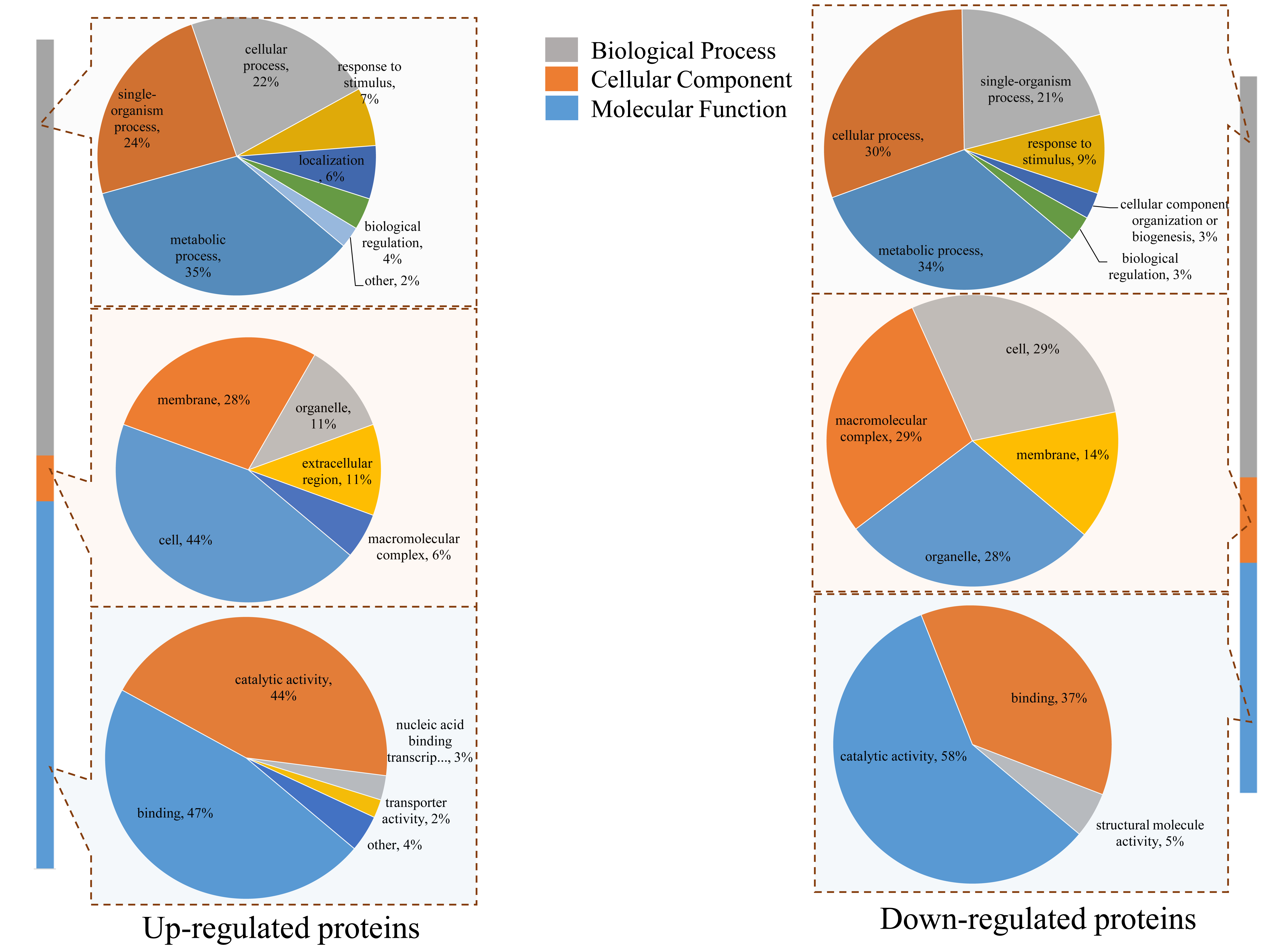

Supplement: Supplemental Information 1 — Distribution of the up-regulated (a) and down-regulated (b) proteins with GO analysis. [file peerj-07-8020-s001.png]

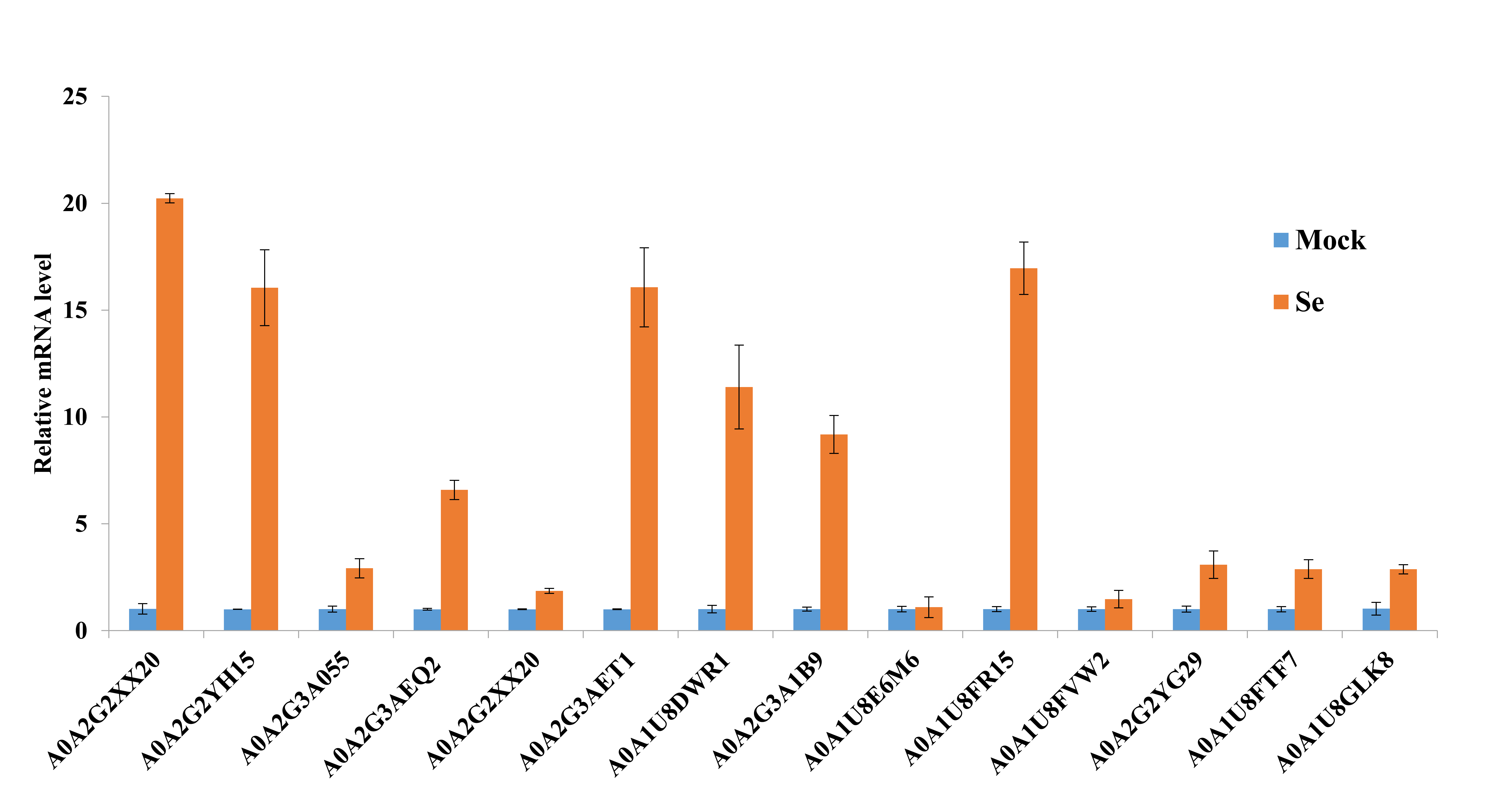

Supplement: Supplemental Information 2 — The data were analyzed by three independent repeats, and standard deviations were shown with error bars. [file peerj-07-8020-s002.png]
